# Utilizing CD30 expression as a rational target for therapy of lymphoma

**DOI:** 10.1186/1756-8722-5-S1-A2

**Published:** 2012-04-25

**Authors:** Won Seog Kim

**Affiliations:** 1 Division of Hematology-Oncology, Sungkyunkwan Univ. School of Medicine, Samsung Medical Center, Seoul, Korea

## Introduction

Rituximab, identified through pivotal lymphoma research, was the first monoclonal antibody approved by the US FDA in 1997. Since the success of rituximab, monoclonal antibodies have been a major focus for development of targeted agents for lymphoma treatment. A major hurdle in the development of a new antibody is finding a new target antigen. CD30 is an attractive therapeutic target antigen, because it has been identified as a marker of Reed–Sternberg cells in Hodgkin lymphoma (HL) [1], it is known to be expressed on anaplastic large cell lymphoma (ALCL), some cases of mediastinal large cell lymphoma, primary effusion lymphoma and multiple myeloma. However, its expression on normal tissues is restricted to a small number of activated B- and T-lymphocytes [2]. Thus, based on its expression pattern, CD30 could be an ideal therapeutic target.

## Naked CD30 targeting antibodies and modified/engineered anti-CD30 antibodies

Although CD30 is considered an ideal target, the results from early clinical trials with first-generation naked monoclonal antibodies targeting CD30 have been disappointing.

Iratumumab (MDX-60) is a fully humanized anti-CD30 monoclonal antibody. Of 72 patients with HL or ALCL, clinical responses were observed in 6 (4 complete response [CR] and 2 partial response [PR]) [3]. SGN-30 is a chimeric anti-CD30 monoclonal antibody. In a phase I trial, one CR was reported in a patient with cutaneous ALCL [4]. In a phase II trial for HL or ALCL, 7 responses (2 CR, 5 PR) in patients with ALCL were reported in 79 patients. Unfortunately, there were no responders in the 38 HL patients [5].

XmAb2513 is a modified anti-CD30 antibody with increased binding affinity to the Fc receptor. In vitro data showed more potent and efficacious cell killing than first-generation anti-CD30 monoclonal antibodies XDA-060 and SGN-30. In clinical trials, only phase I data are available. Therefore, the data are insufficient to assess whether the response is better than that with unmodified antibodies [6].

## Radioimmunoconjugation

Radiation itself is quite an efficient tool for killing lymphoma cells. If a good radiation source, a well-targeted monoclonal antibody and a conjugation technique are available, a radioimmunotherapeutic agent can be a good option in lymphoma treatment. Already radioimmunoconjugations using diverse radionuclides (including ^90^Y and ^131^I) are being investigated. In a preclinical animal model, the survival of mice was significantly prolonged by treatment with anti-CD30 antibody HeFi-1 coupled to ^90^Y [7]. The novel anti-CD30 monoclonal antibody Ki-4 conjugated with ^131^I (total dose 0.0035–0.99 Gy) was trialed in 22 patients with refractory or relapsed HL. One CR, 5 PRs, and 3 minor responses were achieved. However, 7 patients experienced grade 4 hematologic toxicity 4 to 8 weeks after treatment. Therefore, the development of this drug did not continue [8].

## Antibody–drug conjugate: brentuximab–vedotin (SGN-35)

Brentuximab–vedotin (SGN-35) is an anti-CD30 monoclonal antibody conjugated to monomethyl auristatin E (MMAE), a synthetic antitubulin agent. Through binding with CD30, brentuximab–vedotin is internalized. Inside the lysosomes of lymphoma cells, free MMAE is released. Therefore, no immune response is required to achieve efficacy [9].

Two phase I trials with different schedules (treatment every 3 weeks or weekly) have been conducted. In the schedule with treatment every 3 weeks, the maximal tolerated dose was 1.8 mg/kg. Objective responses were observed in 17 of 45 relapsed or refractory CD30-positive hematologic malignancies including HL and ALCL. Of the responders, 11 achieved CR [10]. In a phase II trial with 102 patients with relapse after autologous stem cell transplantation (ASCT), who were treated with 1.8 mg/kg every 3 weeks, the overall response was around 75% (32% CR) with a median duration of response of 6.7 months. In a single arm phase II trial including 58 relapsed systemic ALCL patients, the overall response was 86% (58% CR), with a median duration of response of 12.6 months.

Based on these excellent outcomes, brentuximab–vedotin was approved by the FDA in August 2011. In HL patients, brentuximab–vedotin is indicated after the failure of ASCT or after the failure of at least two prior regimens of combination chemotherapy if the patients are not ASCT candidates. In systemic ALCL patients, it is approved after failure of at least one prior multiagent chemotherapy.

## Overcoming the limitations of anti-CD30 targeting antibodies

Based on its expression patterns, CD30 should be a good therapeutic target. However, it can be shed in a soluble form, resulting in a reduction in the effect of anti-CD30 monoclonal antibodies by competitive binding. Thus, developing a monoclonal antibody targeting membrane-associated CD30 epitopes (Ep2: amino acids 107–153, Ep7 amino acids 282–338) may have potential advantages [11]. Although antibodies have exquisite selectivity for tumor over normal tissue, antibody localization to tumors is inefficient. Diabodies (50–55 kDa) can penetrate tumor more rapidly and accumulate more drug in tumors because they are smaller than IgG (150 kDa). An anti-CD30 diabody–drug conjugate (diabody-vcF4) showed potent antitumor activity and tolerable toxicity in a mouse model [12].

## Future directions

After the early success of anti-CD30 monoclonal antibodies, a variety of clinical trials are ongoing. These antibodies can be combined as a part of first-line treatment, for example, combining ABVD with different levels of SGN-35, as part of salvage combination chemotherapy, maintenance, or as part of a conditioning regimen. After more information is obtained through clinical trials in the near future, new therapeutic strategies can be defined.

## References

[B1] Schwab U Stein H Gerdes J Lemke H Kirchner H Schaadt M Diehl V Production of a monoclonal antibody specific for Hodgkin and Sternberg-Reed cells of Hodgkin's disease and a subset of normal lymphoid cells Nature 1982 299 65 67 10.1038/299065a0 7110326

[B2] Younes A Kadin ME Emerging applications of the tumor necrosis factor family of ligands and receptors in cancer therapy J Clin Oncol 2003 21 3526 3534 10.1200/JCO.2003.09.037 12972530

[B3] Ansell SM Horwitz SM Engert A Khan KD Lin T Strair R Keler T Graziano R Blanset D Yellin M Fischkoff S Assad A Borchmann P Phase I/II study of an anti-CD30 monoclonal antibody (MDX-060) in Hodgkin's lymphoma and anaplastic large-cell lymphoma J Clin Oncol 2007 25 2764 2769 10.1200/JCO.2006.07.8972 17515574

[B4] Bartlett NL Younes A Carabasi MH Forero A Rosenblatt JD Leonard JP Bernstein SH Bociek RG Lorenz JM Hart BW Barton J A phase 1 multidose study of SGN-30 immunotherapy in patients with refractory or recurrent CD30+ hematologic malignancies Blood 2008 111 1848 1854 10.1182/blood-2007-07-099317 18079362 PMC2275000

[B5] Forero-Torres A Leonard JP Younes A Rosenblatt JD Brice P Bartlett NL Bosly A Pinter-Brown L Kennedy D Sievers EL Gopal AK A Phase II study of SGN-30 (anti-CD30 mAb) in Hodgkin lymphoma or systemic anaplastic large cell lymphoma Br J Haematol 2009 146 171 179 10.1111/j.1365-2141.2009.07740.x 19466965

[B6] Blum KA Smith M Fung H Zalevsky J Combs D Ramies DA Younes A Phase I study of an anti-CD30 Fc engineered humanized monoclonal antibody in Hodgkin lymphoma (HL) or anaplastic large cell lymphoma (ALCL) patients: Safety, pharmacokinetics (PK), immunogenicity, and efficacy Journal of Clinical Oncology 2009 27

[B7] Zhang M Yao Z Patel H Garmestani K Zhang Z Talanov VS Plascjak PS Goldman CK Janik JE Brechbiel MW Waldmann TA Effective therapy of murine models of human leukemia and lymphoma with radiolabeled anti-CD30 antibody, HeFi-1 Proc Natl Acad Sci U S A 2007 104 8444 8448 10.1073/pnas.0702496104 17488826 PMC1895969

[B8] Schnell R Dietlein M Staak JO Borchmann P Schomaecker K Fischer T Eschner W Hansen H Morschhauser F Schicha H Diehl V Raubitschek A Engert A Treatment of refractory Hodgkin's lymphoma patients with an iodine-131-labeled murine anti-CD30 monoclonal antibody J Clin Oncol 2005 23 4669 4678 10.1200/JCO.2005.09.098 16034043

[B9] Doronina SO Toki BE Torgov MY Mendelsohn BA Cerveny CG Chace DF DeBlanc RL Gearing RP Bovee TD Siegall CB Francisco JA Wahl AF Meyer DL Senter PD Development of potent monoclonal antibody auristatin conjugates for cancer therapy Nat Biotechnol 2003 21 778 784 10.1038/nbt832 12778055

[B10] Younes A Bartlett NL Leonard JP Kennedy DA Lynch CM Sievers EL Forero-Torres A Brentuximab vedotin (SGN-35) for relapsed CD30-positive lymphomas N Engl J Med 2010 363 1812 1821 10.1056/NEJMoa1002965 21047225

[B11] Nagata S Ise T Onda M Nakamura K Ho M Raubitschek A Pastan IH Cell membrane-specific epitopes on CD30: Potentially superior targets for immunotherapy Proc Natl Acad Sci U S A 2005 102 7946 7951 10.1073/pnas.0502975102 15905329 PMC1142388

[B12] Kim KM McDonagh CF Westendorf L Brown LL Sussman D Feist T Lyon R Alley SC Okeley NM Zhang X Thompson MC Stone I Gerber HP Carter PJ Anti-CD30 diabody-drug conjugates with potent antitumor activity Mol Cancer Ther 2008 7 2486 2497 10.1158/1535-7163.MCT-08-0388 18723494

